# Reinforcement learning personalized instruction for psychological engagement self-regulated learning motivation and performance in professional English education

**DOI:** 10.3389/fpsyg.2026.1900245

**Published:** 2026-08-10

**Authors:** Li Peiyao

**Affiliations:** School of Foreign Languages, Sichuan University, Chengdu, China

**Keywords:** adaptive learning, motivation, personalized learning, professional English education, psychological engagement, reinforcement learning, self-regulated learning

## Abstract

Adaptive educational technologies are increasingly used to personalize learning pathways. However, limited evidence has examined whether reinforcement learning (RL)-personalized instruction is associated with psychologically meaningful learning outcomes in Professional English education. This study compared an RL-personalized instructional pathway with a fixed-path control pathway among 200 learners across an 8-week, 16-session Professional English program. Student-level data included baseline characteristics, English proficiency, psychological engagement, self-regulated learning, motivation, user experience, and performance outcomes. In contrast, session-level data captured learner states, instructional actions, policy type, reward values, difficulty, task category, accuracy, response time, task completion, time on task, and momentary engagement process scores. The groups were comparable at baseline in demographic characteristics and English proficiency. The RL-personalized group showed higher process-level learning activity, including higher reward values, greater exposure to difficulty, more completed tasks, longer time on task, shorter response times, higher task accuracy, and higher momentary engagement process scores. Post-intervention outcomes also favored the RL-personalized group for psychological engagement, self-regulated learning, motivation, user experience, gain score, and final achievement. Adjusted models showed that these associations remained after controlling for baseline English proficiency and participant-level covariates. Because baseline levels of psychological engagement, self-regulated learning, and motivation were not measured, psychological outcomes were interpreted as adjusted post-intervention between-group differences rather than as within-person improvement. These findings suggest that RL-personalized Professional English pathways may support psychologically responsive adaptive instruction when learner-state estimation, difficulty calibration, feedback, professional task relevance, and support for self-regulation are integrated into the learning design.

## 1 Introduction

Artificial intelligence (AI) has become an increasingly influential component of contemporary educational technology, particularly in contexts where learners require individualized support, adaptive feedback, and flexible learning trajectories. Recent reviews of artificial intelligence in education indicate that AI-based systems are no longer limited to automated assessment or tutoring but increasingly support personalization, prediction, feedback generation, learner modeling, and adaptive learning design (Maghsudi et al., 2021; Zawacki-Richter et al., 2019; Chen et al., 2020; Zhai et al., 2021; Zafari et al., 2022). In language education, these developments are especially relevant because learners differ substantially in prior proficiency, task-specific weaknesses, communication goals, motivation, and self-regulatory capacity. Professional English education further intensifies this challenge because instructional tasks are expected to support not only general linguistic proficiency but also workplace-oriented communication, professional writing, presentation skills, and case-based interaction.

Personalized learning has been proposed as a response to learner heterogeneity in digitally mediated education. Conceptually, personalized learning refers to instructional adaptation based on learner needs, prior knowledge, performance, preferences, and progression states (Shemshack and Spector, 2020). Adaptive learning can be viewed as a more dynamic form of personalization, in which instructional decisions are repeatedly updated in response to learner performance, behavior, and feedback during learning. Empirical and review evidence suggests that technology-supported personalized learning is most useful when adaptation is linked to meaningful instructional decisions rather than superficial content variation (Xie et al., 2019; Martin et al., 2020; Major et al., 2021). However, personalized learning is often implemented through rule-based pathways, static learner profiles, or limited recommendation mechanisms. Fewer studies examine adaptive systems that continuously update instructional decisions based on learner behavior and feedback over time (Xie et al., 2019; Martin et al., 2020; Den Hengst et al., 2020). This limitation is particularly important in Professional English education, where learners’ needs evolve dynamically as they move across vocabulary, reading, writing, speaking, and professional communication tasks.

Reinforcement learning (RL) provides a theoretically sound framework for dynamic personalization because it enables an instructional system to select actions, observe learner responses, receive reward feedback, and update its future decisions. In educational settings, RL can support task sequencing, feedback timing, difficulty adjustment, intervention selection, and individualized learning pathways (Den Hengst et al., 2020; Memarian and Doleck, 2024). Broader reviews of RL-based personalization show that RL has been increasingly applied in human-centered systems requiring decisions to be adapted to users’ states and outcomes (Den Hengst et al., 2020). More recent education-focused reviews also indicate growing interest in RL for tutoring systems, adaptive learning environments, and personalized educational interventions (Memarian and Doleck, 2024). Nevertheless, existing RL-in-education studies remain more concentrated on technical optimization, policy selection, and performance prediction than on psychological learning processes. Therefore, an important unresolved question is whether RL-personalized instruction is associated not only with task performance but also with psychological engagement, motivation, and self-regulated learning.

Psychological engagement is central to this question because it reflects cognitive investment, emotional involvement, and behavioral participation in learning. Recent learning analytics research has shown that engagement is often operationalized through observable behavioral traces, such as clicks, time on task, logins, and task completion, while multidimensional psychological engagement is less frequently captured (Bergdahl et al., 2024). Broader evidence maps and systematic reviews of educational technology in higher education also show that engagement is a complex, multidimensional construct that can be supported by technology but requires careful conceptualization and measurement (Bond et al., 2020; Bedenlier et al., 2020). For this reason, research on AI-supported personalization should not rely solely on behavioral analytics. It should also integrate psychological engagement indicators with learning-log data to clarify how adaptive systems are associated with both observable participation and internal learning involvement.

Self-regulated learning is also relevant because adaptive educational systems may support learners not only by selecting content but also by scaffolding goal setting, planning, monitoring, and revision. In online and blended learning environments, learners must regulate their effort, monitor their progress, and adjust their strategies across tasks. Systematic reviews show that learning analytics interventions can support self-regulated learning by providing feedback, monitoring information, and actionable prompts (Heikkinen et al., 2023). Meta-analytic evidence further indicates that training in self-regulated learning is associated with stronger academic performance, self-regulated learning strategies, and motivation among university students (Theobald, 2021). A more recent meta-analysis in online higher education also confirms that self-regulated learning strategies are positively associated with academic performance (Cheng et al., 2025). These findings provide a rationale for examining self-regulated learning as a core psychological outcome in RL-personalized instruction.

Motivation represents a third relevant construct because it influences persistence, learning interest, perceived usefulness, and willingness to continue engaging with demanding instructional tasks. In language learning, AI-supported and technology-enhanced environments may be associated with motivation when they provide immediate feedback, individualized pacing, interactive practice, and task relevance (Qiao and Zhao, 2023; Rahmati et al., 2021; Hasumi and Chiu, 2024; Seyyedrezaei et al., 2024). A recent study in an EFL context showed that AI-based language learning was associated with speaking skills and self-regulation, indicating that AI-supported instruction may relate to both language performance and psychological learning processes (Qiao and Zhao, 2023). Evidence from English language teaching and technology-enhanced language learning also highlights the importance of digital tools (Rahmati et al., 2021), feedback, and learner-centered support in language education (Hasumi and Chiu, 2024; Seyyedrezaei et al., 2024). However, relatively little empirical work has examined RL-personalized Professional English instruction as a pathway that jointly addresses psychological engagement, self-regulated learning, motivation, user experience, and performance.

Learning analytics research provides another foundation for the present study. Analytics-based feedback has been shown to support engagement in online learning (Karaoglan Yilmaz and Yilmaz, 2022) and self-regulated learning and academic achievement in blended EFL courses (Chen, 2023). These findings suggest that data-informed instructional feedback can be linked to learner behavior and learning outcomes. However, many learning analytics interventions remain feedback-oriented rather than policy-oriented: they inform learners or instructors but do not continuously adapt the instructional pathway. RL-personalized learning extends this approach by embedding learner analytics directly into instructional decision-making, where learner state, instructional action, difficulty, recommendation type, and reward feedback inform an adaptive policy.

Several gaps remain. AI and personalized-learning studies often prioritize achievement or system performance, with less attention to psychological engagement and self-regulated learning. RL applications also remain limited in language-learning contexts, particularly in Professional English education. Moreover, behavioral logs and self-report outcomes are often examined separately rather than integrated with learner states, interaction patterns, psychological outcomes, and performance indicators. Evidence is also limited regarding whether RL-personalized instruction is associated with both subjective learning experience and adjusted performance outcomes after accounting for baseline equivalence.

This study therefore examined RL-personalized instruction in Professional English education, focusing on psychological engagement, self-regulated learning, motivation, user experience, and English performance. Using a two-group design with 200 learners and 16-session interaction records, the study compared an RL-personalized pathway with a fixed control pathway. The analysis integrated student-level psychological and performance outcomes with interaction-level indicators, including learner state, action, reward, difficulty, task type, login frequency, time on task, task completion, response time, and momentary engagement. The study addresses the following aims:

- To evaluate an RL-personalized instructional pathway in a Professional English education context.
- To examine post-intervention differences in psychological engagement, self-regulated learning, motivation, user experience, and English performance between the RL-personalized and fixed-path conditions.
- To combine student-level and session-level data in order to describe both overall outcome differences and learning-interaction trajectories across 16 instructional sessions.
- To operationalize personalization through learner states, instructional actions, policy type, task difficulty, recommended task categories, and reward feedback.
- To clarify whether adaptive personalization is associated with psychologically meaningful learning processes in addition to achievement-related outcomes.

## 2 Materials and methods

### 2.1 Study design, setting, and design rationale

This study used a two-arm, parallel-group, controlled intervention design to compare an RL-personalized Professional English pathway with a fixed-path control pathway with respect to psychological engagement, self-regulated learning, motivation, user experience, and English performance. The study was conducted from September 15 to December 20, 2025. The eight-week intervention was delivered from October 1 to November 26, 2025, and comprised 16 structured sessions per learner.

Participants were assigned in a 1:1 ratio to the RL-personalized or fixed-path control condition. The RL-personalized group received adaptive recommendations based on learner-state estimates, prior performance, task difficulty, task category, and reward feedback. The control group used the same platform, content bank, session schedule, and assessment structure but followed a fixed non-adaptive sequence.

**Figure 1:**
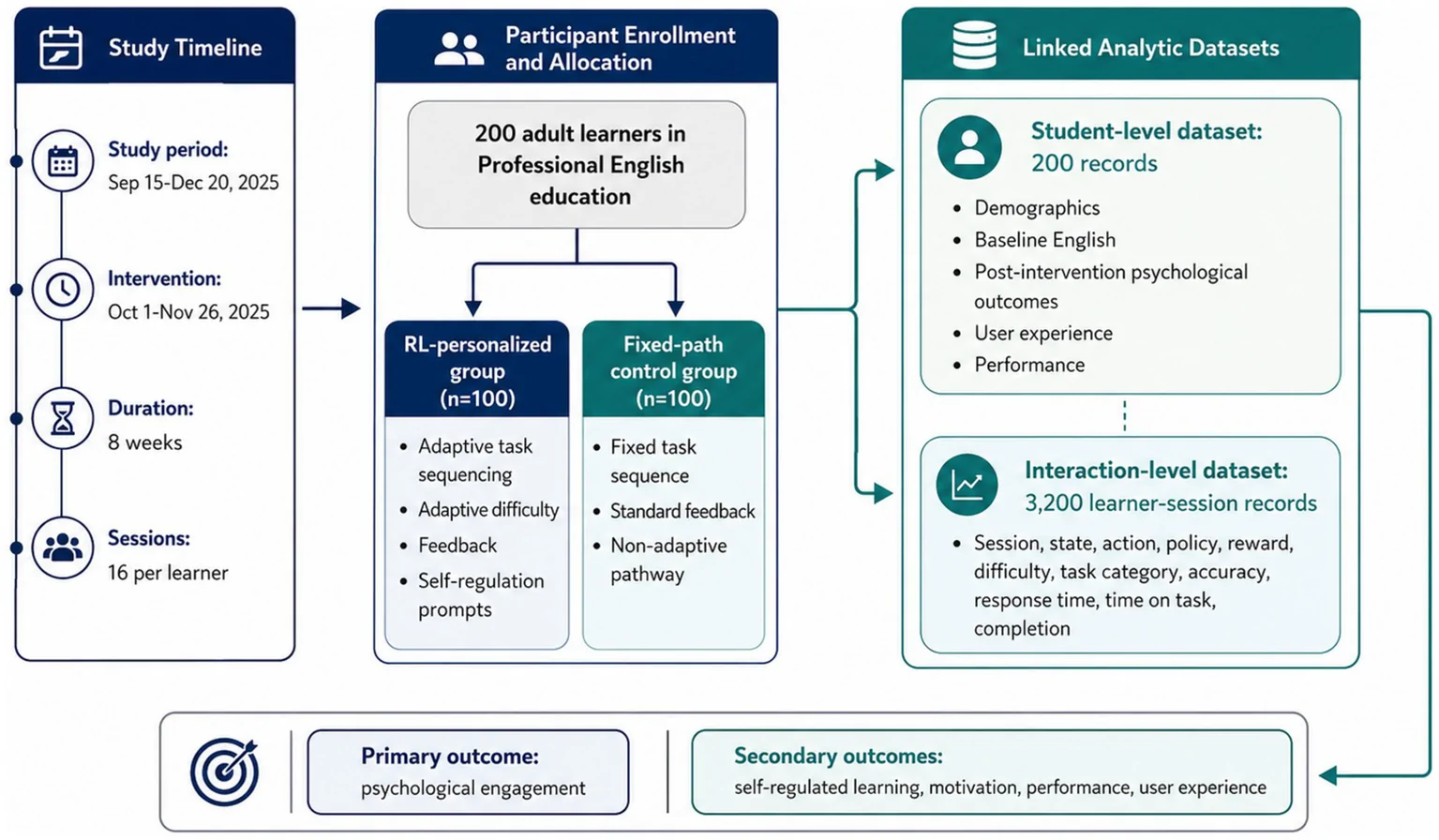
Study design, participant allocation, instructional conditions, and linked student-level/session-level dataset structure.

The study therefore compared an RL-personalized instructional pathway with a fixed-path pathway. Because the personalized condition also involved adaptive difficulty, feedback, sequencing, and self-regulation prompts, the analyses estimate group-level intervention differences rather than the isolated causal effect of the Q-learning algorithm. The study workflow, allocation, instructional conditions, and linked data structure are summarized in Figure 1, and the main design and internal-validity features are presented in Table 1.

**Table 1:** Study design, allocation, and internal-validity features.

Design element	Methodological specification
Study type	Two-arm, parallel-group controlled intervention study
Study period	September 15 to December 20, 2025
Intervention period	October 1 to November 26, 2025
Intervention duration	Eight weeks
Number of sessions	16 structured learning sessions per learner
Allocation ratio	1:1 allocation to RL-personalized and fixed-path control conditions
Final analytic sample	200 learners; 100 in the RL-personalized group and 100 in the control group
Session-level records	3,200 learner-session records, generated as 200 learners × 16 sessions
Primary outcome	Post-intervention total psychological engagement
Secondary psychological outcomes	Self-regulated learning score and motivation score
Secondary performance outcomes	Post-test English score, gain score, task accuracy percentage, and final achievement
User-experience outcomes	Satisfaction, perceived personalization, and perceived feedback quality
Blinding	Participants and instructors could not be blinded because the instructional pathways were visibly different. Group labels were coded during data cleaning and statistical modeling to reduce analyst-side interpretive bias where feasible.
Baseline psychological measures	Baseline English proficiency was measured before the intervention. Baseline psychological engagement, self-regulated learning, and motivation were not included; therefore, post-intervention psychological outcomes were interpreted as adjusted between-group differences.
Internal-validity qualification	The study compares an RL-personalized instructional pathway with a fixed-path pathway. It does not fully separate the effect of the RL algorithm from co-occurring adaptive features such as feedback, difficulty calibration, self-regulation prompts, task sequencing, and learning dosage.

### 2.2 Participants, recruitment, allocation, and consent

The final analytic dataset included 200 adult learners enrolled in a Professional English program. Eligible learners were enrolled in the course/module, able to complete the eight-week digital program, completed the baseline English proficiency assessment, and provided informed consent for research use of anonymized questionnaire, performance, and learning-log data. Learners were excluded if they declined consent, missed baseline assessment, withdrew before the first session, or lacked identifiers required for student- or session-level linkage.

Participants were recruited through course announcements and learning management system notices. Participation was voluntary; no financial incentive was provided; and learners were informed that declining to use research data would not affect course access, academic standing, or access to instructional materials. Before the intervention, learners were individually assigned to conditions using a computer-generated 1:1 sequence, with coded group assignments recorded prior to the learning-log export. Baseline comparability was assessed across demographic variables, prior English score, overall pre-test score, and domain-specific pre-test scores. Participant and instructor blinding was not feasible because the fixed and adaptive pathways were visibly different.

The cohort was selected because Professional English education is well-suited to adaptive personalization, given the variation in proficiency, academic level, disciplinary background, and professional communication needs. The sample included undergraduate, master’s-level, and professional-certificate learners from business/management, engineering, health sciences, social sciences, computer science, and other fields.

### 2.3 Dataset structure and linkage

Two linked datasets were analyzed. The student-level dataset contained one record per learner. It included demographics, prior English score, baseline English proficiency, post-intervention psychological engagement, self-regulated learning, motivation, user experience, learning-log aggregates, and English performance outcomes.

The interaction-level dataset contained one record per learner-session interaction, including session number, learner state, instructional action, policy type, reward, task difficulty, recommended task category, task accuracy, response time, task completion, time on task, and momentary engagement. Each learner contributed 16 session records, yielding 3,200 learner-session observations. Student- and interaction-level records were linked using anonymized student ID and case number.

No delayed follow-up dataset was available; therefore, the analyses focused on immediate post-intervention psychological, user experience, interaction, and performance outcomes rather than on long-term retention or durability. The linked data structure is summarized in Table 2.

**Table 2:** Structure of the linked analytic datasets.

Dataset	Unit of analysis	Number of records	Key variables	Analytical role
Student-level dataset	Learner	200	Demographics, prior English score, pre-test English score, psychological engagement, self-regulated learning, motivation, user experience, post-test score, gain score, task accuracy percentage, final achievement	Used for baseline equivalence, primary outcome analysis, secondary psychological outcomes, performance outcomes, user-experience outcomes, adjusted linear models, and correlation analyses
Interaction-level dataset	Learner-session	3,200	Session number, learner state, instructional action, policy type, reward value, difficulty level, recommended task category, task accuracy, response time, task completion, time on task, momentary engagement	Used for learning-interaction trajectories, mixed-effects models, Group × Session analyses, process indicators, and exploratory mechanism analyses
Codebook	Variable definition	—	Coding of group, gender, academic level, major, learner state, action, policy, difficulty, recommended task, and scale ranges	Used for reproducible coding, range checks, and interpretation of categorical variables

### 2.4 Professional English learning program and instructional comparability

The program focused on academic and workplace-oriented Professional English within an English for Specific Purposes framework. Activities covered five task families: vocabulary development, reading comprehension, professional writing/email communication, speaking and presentation practice, and case-based professional communication.

Both groups used the same platform, content bank, schedule, and assessment structure. The key contrast was the task-selection rule. The control group followed a fixed Professional English sequence, whereas the RL-personalized group received adaptive recommendations based on learner state, prior performance, task category, difficulty, and reward feedback. Adaptive actions could include remediation feedback, reinforcement practice, increased difficulty, scenario-based professional tasks, or self-regulation prompts.

**Figure 2:**
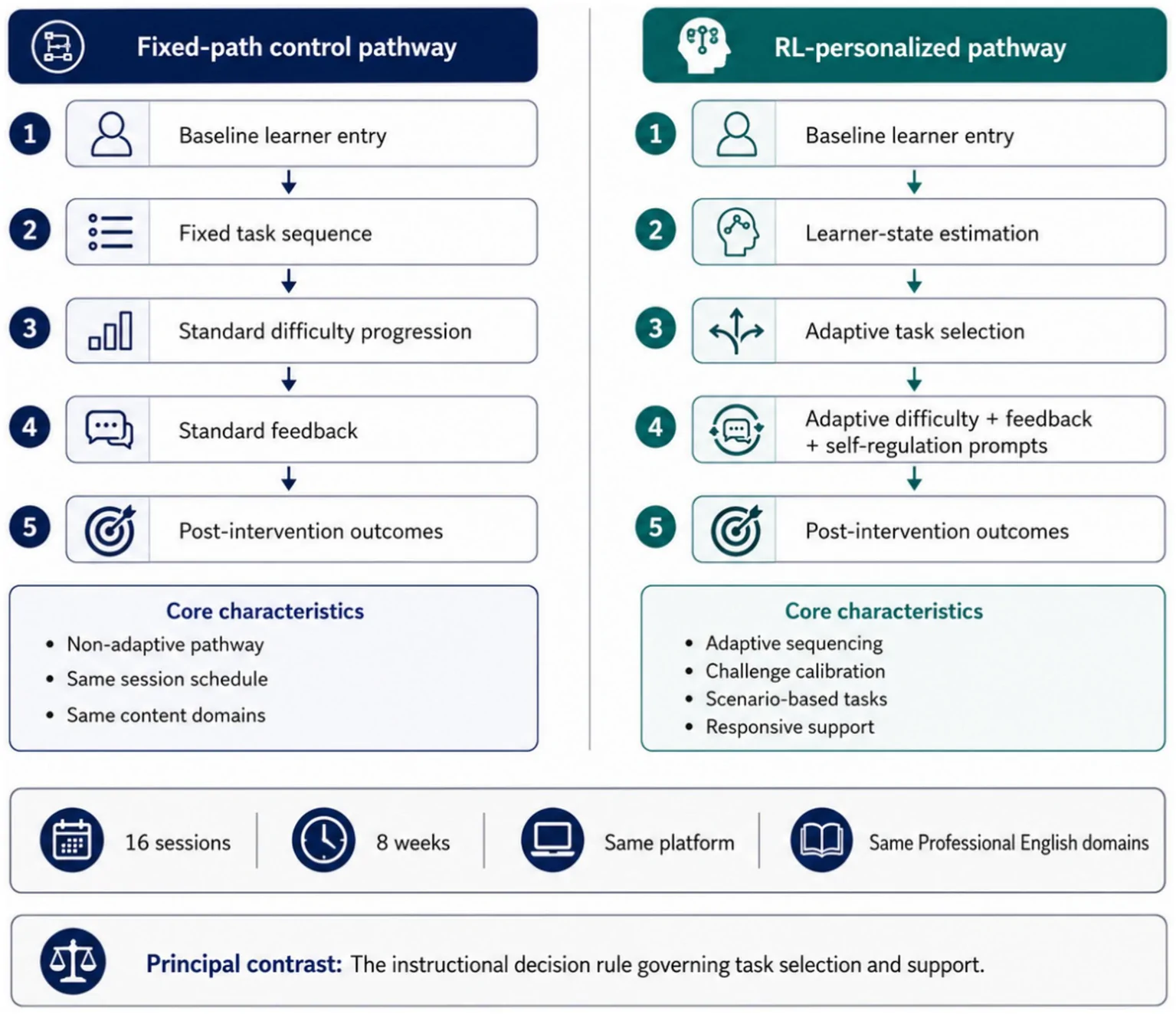
Fixed-path control pathway versus reinforcement learning-personalized professional English pathway.

Comparability was supported by aligning session number, content domains, platform access, and assessment timing across groups. Differences in dosage, completed tasks, time on task, feedback exposure, and difficulty exposure were treated as process indicators and analyzed rather than assumed equivalent. Figure 2 illustrates the two instructional pathways, and Table 3 summarizes content, comparability controls, and fidelity monitoring.

**Table 3:** Instructional program, comparability features, and fidelity monitoring.

Component	Fixed-path control condition	RL-personalized condition	Fidelity/Comparability approach
Program focus	Professional English for academic and workplace communication	Professional English for academic and workplace communication	Same broad instructional domain
Session schedule	16 sessions across 8 weeks	16 sessions across 8 weeks	Same planned number of sessions
Content domains	Vocabulary, reading, writing/email, speaking/presentation, case-based communication	Vocabulary, reading, writing/email, speaking/presentation, case-based communication	Same content bank and domain taxonomy
Task sequence	Fixed sequence determined before the course	Adaptive sequence selected by RL policy	Sequencing difference was the intended intervention contrast
Task difficulty	Predefined progression	Dynamically adjusted from level 1 to level 5	Difficulty exposure was logged and analyzed
Feedback	Standard fixed-path feedback	Adaptive feedback based on learner state and action selection	Feedback exposure was considered part of the personalized pathway
Self-regulation support	Standard learning instructions	Self-regulation prompts could be selected as an instructional action	Prompt exposure was logged as part of action data
Platform access	Same platform and course access	Same platform and course access	Platform-level logs verified access and session completion
Fidelity indicators	Login frequency, time on task, completed tasks, attempts, session completion	Login frequency, time on task, completed tasks, attempts, session completion, adaptive action logs	Fidelity was monitored through automated logs, session records, and coding checks

### 2.5 Reinforcement learning personalization framework

The RL framework was implemented as a constrained tabular Q-learning procedure embedded in the Professional English platform. Because each learner completed only 16 sessions, the system did not train a high-dimensional learner-specific policy from scratch. Instead, it used a pedagogically constrained state-action structure, initialized with instructional priors and updated using learner-session experience across the intervention group.

The framework included learner state, composite instructional action, policy type, reward value, and policy update. Learner state represented the pre-action instructional condition and was estimated from mastery-related and process-engagement indicators available before the next task. It did not use the post-intervention psychological engagement questionnaire and was therefore analytically distinct from the primary outcome.

**Figure 3:**
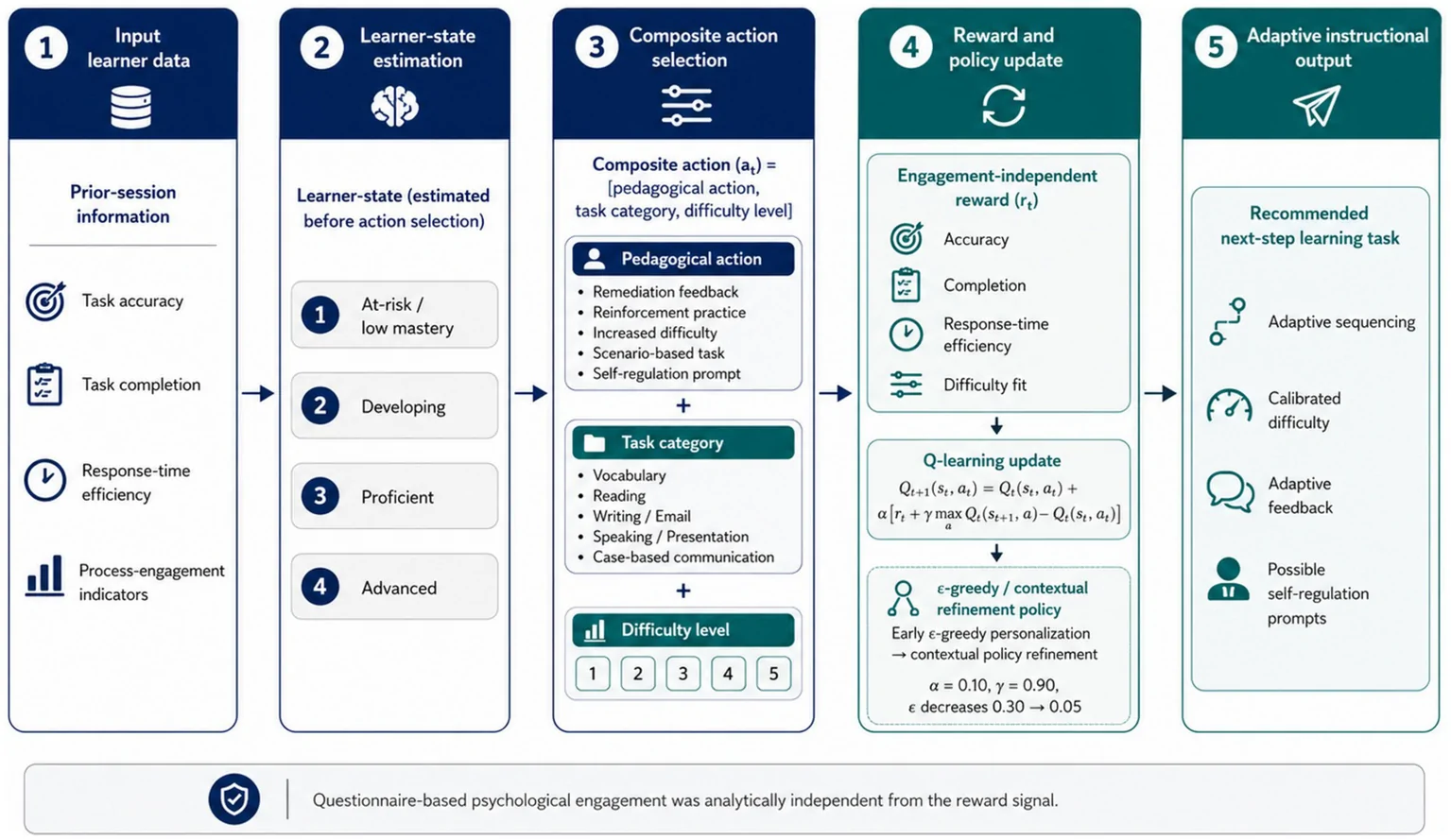
Reinforcement learning personalization pipeline: learner-state estimation, composite action selection, engagement-independent reward construction, and policy updating.

The composite action space jointly included pedagogical action, recommended Professional English task category, and task difficulty level. Thus, each instructional action represented a combined decision about support type, content category, and difficulty assignment. The RL personalization pipeline is summarized in Figure 3, and the operational RL components are defined in Table 4.

**Table 4:** Operational structure of the reinforcement learning personalization framework.

Component	Coding or scale	Operational definition	Analytical role
Learner state	1 = at-risk/low mastery; 2 = developing; 3 = proficient; 4 = advanced	Estimated before action selection using prior-session mastery indicators, task accuracy, task completion, response-time efficiency, and process-engagement indicators. It did not use the post-intervention psychological engagement questionnaire.	Defines the learner condition before instructional action selection
Pedagogical action	0 = fixed-control module; 1 = remediation feedback; 2 = reinforcement practice; 3 = increased difficulty; 4 = scenario-based professional task; 5 = self-regulation prompt	The pedagogical support decision selected for the next learning step	Defines the instructional support strategy
Recommended task category	1 = vocabulary; 2 = reading comprehension; 3 = professional writing/email; 4 = speaking/presentation; 5 = case-based professional communication	The Professional English task family selected for the learner	Defines the content domain
Difficulty level	1 to 5	Assigned task difficulty level	Defines challenge calibration
Composite action	*a*_*it* = {pedagogical action, task category, difficulty level}	Joint instructional decision combining support type, task category, and difficulty	Defines the actionable personalization unit
Policy type	0 = fixed control path; 1 = early epsilon-greedy *Q* personalization; 2 = contextual policy refinement	Decision strategy used for task selection	Defines the learning-path selection strategy
Reward value	−1 to +1	Normalized engagement-independent reward based on task accuracy, task completion, response-time efficiency, and difficulty fit	Defines the policy-updating signal
Momentary engagement	1 to 5 session-level process indicator	Logged as a session-level process indicator. It was not included in the revised reward formula and was not treated as equivalent to the primary questionnaire-based psychological engagement outcome.	Used as a process descriptor

#### 2.5.1 Learner-state estimation

For learner *i* at session *t*, learner state was estimated before selection of the next instructional action. The state estimate used information available up to the previous task or session, including rolling task accuracy, task completion history, response-time efficiency, and process-engagement indicators. This temporal ordering was used to avoid leakage from post-action outcomes into the pre-action state estimate. A continuous learner-readiness score was calculated using Equation 1:


$$R_{\mathrm{it}}=0.50A_{i,t-1}+0.20C_{i,t-1}+0.15E_{i,t-1}+0.15T_{i,t-1}$$ (1)

where $A_{i,t-1}$ is normalized rolling task accuracy, $C_{i,t-1}$ is recent task completion, $E_{i,t-1}$ is process-engagement information available before the action, and $T_{i,t-1}$ is response-time efficiency. The score was discretized into four pedagogically interpretable states using Equation 2:


$${\text{State}}_{\mathrm{it}}=\{\begin{array}{cc} 1, & R_{\mathrm{it}}<0.50\\ 2, & 0.50\leq R_{\mathrm{it}}<0.65\\ 3, & 0.65\leq R_{\mathrm{it}}<0.80\\ 4, & R_{\mathrm{it}}\geq 0.80 \end{array}$$ (2)

The four-state structure was used to preserve interpretability and to prevent the state-action table from becoming sparse across only 16 instructional sessions per learner.

#### 2.5.2 Engagement-independent reward function

To avoid circularity between reward construction and the primary psychological engagement outcome, the reward function used for policy updating did not include the post-intervention psychological engagement questionnaire. It also did not use the total psychological engagement score as an algorithmic input. The reward was based on proximal learning-interaction indicators: task accuracy, task completion, response-time efficiency, and difficulty fit. Momentary engagement was retained as a descriptive process indicator, but questionnaire-based psychological engagement was kept analytically independent from the reward signal. For learner i at session t , For learner *i* at session t, the raw reward score was calculated using Equation 3:


$${\mathrm{RR}}_{\mathrm{it}}=0.40{\mathrm{Acc}}_{\mathrm{it}}+0.25{\text{Comp}}_{\mathrm{it}}+0.20{\mathrm{RTE}}_{\mathrm{it}}+0.15{\mathrm{DF}}_{\mathrm{it}}$$ (3)

where ${\mathrm{RR}}_{\mathrm{it}}$ denotes raw reward, ${\mathrm{Acc}}_{\mathrm{it}}$ is normalized task accuracy, ${\text{Comp}}_{\mathrm{it}}$ is task completion coded from 0 to 1, ${\mathrm{RTE}}_{\mathrm{it}}$ is response-time efficiency normalized from 0 to 1, and ${\mathrm{DF}}_{\mathrm{it}}$ indicates whether assigned difficulty was appropriate for the learner state. The raw reward was rescaled to the interval −1 to +1 using Equation 4:


$${\text{Reward}}_{\mathrm{it}}=2\times {\mathrm{RR}}_{\mathrm{it}}-1$$ (4)

This reward definition optimized the policy toward performance-related and interaction-quality indicators without directly embedding the primary psychological engagement outcome into the reward signal.

#### 2.5.3 *Q*-learning update, exploration, and policy transition

The tabular *Q*-value update was specified in Equation 5:


$$Q_{t+1}=Q_{t}+\alpha [r_{t}+\gamma \max_{a}\;Q_{t}(s_{t+1},a)-Q_{t}]$$ (5)

where $Q_{t}$ denotes $Q_{t}(s_{t},a_{t})$ , $s_{t}$ is the learner state at session t , $a_{t}$ is the composite instructional action, $r_{t}$ is the reward value, a is the learning rate, and γ is the discount factor. The learning rate was set at a=0.10 and the discount factor was set at γ=0.90 . During the early personalization phase, action selection followed the epsilon-greedy rule specified in Equation 6:


$$a_{t}=\{\begin{array}{ll} \text{random feasible action} & \text{with probability}\;\epsilon_{t}\\ \arg\max_{a}Q(s_{t},a) & \text{with probability}\;1-\epsilon_{t} \end{array}$$ (6)

The exploration parameter $\epsilon_{t}$ decreased from 0.30 during the initial sessions to 0.05 during the contextual refinement phase. During contextual policy refinement, the feasible action set was further restricted using pedagogical constraints derived from learner state, prior task performance, and Professional English task domain. For example, learners in lower readiness states were preferentially eligible for remediation feedback and reinforcement practice, whereas learners in higher readiness states were eligible for increased difficulty, scenario-based professional tasks, and advanced professional communication activities. The policy transition was defined in Equation 7:


$${\text{Policy}}_{\mathrm{it}}=\{\begin{array}{ll} 0 & \text{fixed}-\text{path control}\\ 1 & \text{epsilon}-\text{greedy}\;Q\;\text{personalization}\\ 2 & \text{contextual policy refinement} \end{array}$$ (7)

Because only 16 sessions were available per learner, the policy was interpreted as a constrained adaptive personalization procedure rather than a fully converged RL policy. The study evaluated the educational pathway generated by this constrained RL-personalized procedure rather than the theoretical optimality of a fully learned policy.

### 2.6 Variables and measurement instruments

The analytic variables included baseline demographic indicators, baseline English proficiency, RL process variables, learning-log indicators, psychological engagement, self-regulated learning, motivation, performance outcomes, and user-experience outcomes. Table 5 summarizes the main variable domains and scale formats.

**Table 5:** Main analytic variables and measurement scales.

Domain	Variables	Scale or coding	Timing
Baseline characteristics	Age, gender code, academic level code, major code, prior English score	Age in years; categorical codes; prior English score 0–100	Baseline
Baseline English proficiency	Pre-test English score, vocabulary pre-test, reading pre-test, writing pre-test, speaking pre-test	0–100	
RL variables	Dominant state code, dominant action code, policy code, mean reward, mean difficulty level, dominant recommended task code	Categorical codes; reward −1 to +1; difficulty 1–5	Session-level and aggregated student-level
Learning-log indicators	Login frequency, time on task, completed tasks, attempts, average response time	Counts, minutes, seconds	During intervention
Psychological engagement	Cognitive engagement, emotional engagement, behavioral engagement, psychological engagement total	1–5 Likert-type scale	Post-intervention
Self-regulated learning	Goal setting, planning, monitoring, revision behavior, self-regulated learning score		
Motivation	Intrinsic motivation, learning interest, perceived usefulness, motivation score		
Final performance	Post-test score, gain score, task accuracy percentage, final achievement	0–100	Post-intervention and aggregated intervention record
User experience	Satisfaction, perceived personalization, perceived feedback quality	1–5 Likert-type scale	Post-intervention

#### 2.6.1 Baseline English proficiency test

Baseline English proficiency was measured before the intervention using a Professional English proficiency test aligned with the instructional content. The test covered four domains: vocabulary, reading comprehension, professional writing/email communication, and speaking/presentation performance. Each domain score was transformed to a 0–100 scale. The overall pre-test English score was calculated as the mean of the four domain scores using Equation 8:


$${\text{Pretest}}_{i}=\frac{{\text{Vocab}}_{i}+{\text{Read}}_{i}+{\text{Write}}_{i}+{\text{Speak}}_{i}}{4}$$ (8)

The post-test English score used the same domain structure and 0–100 scaling. To support content validity, test content was mapped to the Professional English curriculum and reviewed for coverage of vocabulary, reading, professional writing/email, and speaking/presentation competencies.

#### 2.6.2 Psychological engagement

Psychological engagement was assessed as a multidimensional construct comprising cognitive engagement, emotional engagement, and behavioral engagement. Items used a 1–5 Likert-type response format, with higher scores indicating stronger engagement. Cognitive engagement reflected effortful investment, strategic attention, and perceived cognitive involvement. Emotional engagement reflected interest, enjoyment, and affective connection with learning tasks. Behavioral engagement reflected participation, persistence, and active involvement. The total psychological engagement score was calculated using Equation 9:


$${\text{Engagement}}_{i}=\frac{{\text{Cognitive}}_{i}+{\text{Emotional}}_{i}+{\text{Behavioral}}_{i}}{3}$$ (9)

The questionnaire-based psychological engagement score was independent of the RL reward function and was not used to update the RL policy.

#### 2.6.3 Self-regulated learning

Self-regulated learning was measured using four dimensions: goal setting, planning, monitoring, and revision behavior. Items used a 1–5 Likert-type response format, with higher scores indicating stronger self-regulatory behavior. The total self-regulated learning score was calculated using Equation 10:


$${\mathrm{SRL}}_{i}=\frac{{\text{Goal}}_{i}+{\text{Plan}}_{i}+{\text{Monitor}}_{i}+{\mathrm{Rev}}_{i}}{4}$$ (10)

#### 2.6.4 Motivation

Motivation was measured using three dimensions: intrinsic motivation, learning interest, and perceived usefulness. Items used a 1–5 Likert-type response format, with higher scores indicating stronger motivation. The total motivation score was calculated using Equation 11:


$${\text{Motivation}}_{i}=\frac{{\text{Intrinsic}}_{i}+{\text{Interest}}_{i}+{\text{Usefulness}}_{i}}{3}$$ (11)

#### 2.6.5 User experience

User experience was assessed through satisfaction, perceived personalization, and perceived feedback quality. Satisfaction reflected the overall perceived quality of the learning experience. Perceived personalization reflected the extent to which learners perceived the instructional pathway as responsive to their needs and progress. Perceived feedback quality reflected the perceived usefulness, clarity, and timeliness of instructional feedback.

#### 2.6.6 Instrument structure, reliability, and psychometric assessment

Before scale aggregation, item-level questionnaire responses were checked for coding direction, valid response range, and scale membership. Internal consistency was assessed from item-level responses using Cronbach’s alpha and McDonald’s omega. Cronbach’s alpha was calculated from the item covariance matrix, whereas McDonald’s omega was estimated using a one-factor congeneric model with standardized factor loadings. Reliability was estimated for the baseline English proficiency test, psychological engagement, self-regulated learning, motivation, and user-experience scales.

All scales were coded so that higher scores indicated higher levels of the target construct. Psychological, self-regulated learning, motivation, and user-experience items used 1–5 Likert anchors ranging from 1 = strongly disagree to 5 = strongly agree. Item wording was adapted to the Professional English context and reviewed for content alignment before administration. Table 6 summarizes the instrument’s structure and scoring, and Table 7 reports internal consistency estimates.

**Table 6:** Measurement instruments, item structure, scoring, and validity evidence.

Construct/Scale	Item structure	Response anchors	Scoring procedure	Psychometric/validity evidence
Baseline English proficiency	Four domain components: vocabulary, reading, professional writing/email, and speaking/presentation	0–100 domain scores	Overall pre-test = mean of four domain scores; post-test used the same domain structure	Curriculum mapping and expert review supported content validity; internal consistency was estimated before analysis
Psychological engagement	Multidimensional engagement scale with cognitive, emotional, and behavioral dimensions	1 = strongly disagree to 5 = strongly agree	Dimension scores were averaged; total engagement = mean of cognitive, emotional, and behavioral engagement	Item-to-dimension mapping was reviewed before aggregation; reliability was estimated using Cronbach’s alpha and McDonald’s omega
Self-regulated learning	Goal setting, planning, monitoring, and revision behavior dimensions		Total SRL score = mean of four dimension scores	Theoretical dimensional structure was retained; reliability was estimated using Cronbach’s alpha and McDonald’s omega
Motivation	Intrinsic motivation, learning interest, and perceived usefulness dimensions		Total motivation score = mean of three dimension scores	Items were adapted to the Professional English learning context; reliability was estimated using Cronbach’s alpha and McDonald’s omega
User experience	Satisfaction, perceived personalization, and perceived feedback quality indicators		Indicators were analyzed separately and summarized as a user-experience reliability set	Content alignment was reviewed for platform-based learning; reliability was estimated using Cronbach’s alpha and McDonald’s omega

**Table 7:** Internal-consistency reliability estimates calculated from item-level responses before scale aggregation.

Construct/Scale	Indicators used for scale construction	Cronbach’s *α*	McDonald’s *ω*	Interpretation
Baseline English proficiency	Vocabulary pre-test, reading pre-test, writing pre-test, speaking pre-test	0.952	0.966	Excellent
Psychological engagement	Cognitive engagement, emotional engagement, behavioral engagement	0.688	0.829	Marginal by *α*; good by *ω*
Self-regulated learning	Goal setting, planning, monitoring, revision behavior	0.723	0.828	Acceptable/good
Motivation	Intrinsic motivation, learning interest, perceived usefulness	0.739	0.852	Acceptable/good
User experience	Satisfaction, perceived personalization, perceived feedback quality	0.822	0.902	Good/excellent

### 2.7 Outcome definitions

The primary outcome was post-intervention total psychological engagement. Secondary psychological outcomes were self-regulated learning score and motivation score. Secondary performance outcomes were post-test English score, gain score, task accuracy percentage, and final achievement. User-experience outcomes were satisfaction, perceived personalization, and perceived feedback quality. The gain score was calculated using Equation 12:


$${\text{Gain}}_{i}={\text{Posttest}}_{i}-{\text{Pretest}}_{i}$$ (12)

Task accuracy percentage was calculated from the intervention task records using Equation 13:


$$\text{Task}\;{\text{Accuracy}}_{i}=(\frac{\text{Correct}\;{\text{Attempts}}_{i}}{\text{Total}\;{\text{Attempts}}_{i}})\times 100$$ (13)

Final achievement was recorded as the final course-level English achievement indicator on a 0–100 scale after aggregation of post-intervention performance evidence according to the program scoring rubric. Session-level process outcomes included reward value, task accuracy, response time, task completion, time on task, and momentary engagement. Momentary engagement was treated as a process indicator and was not treated as equivalent to questionnaire-based total psychological engagement.

### 2.8 Data management and quality control

Data were organized into student-level and interaction-level files. The student-level dataset included 200 complete learner records, and the interaction-level dataset included 3,200 complete learner-session observations. All records were anonymized with coded identifiers, and student- and session-level data were linked using student ID and case number.

Before analysis, quality-control checks verified group coding, duplicate identifiers, session counts, score ranges, coding consistency, missingness, impossible values, and formula-derived variables. Variables on 0–100 scales, 1–5 Likert scales, categorical codes, and −1 to +1 reward values were screened against predefined ranges. Each learner was expected to contribute 16 session records, which were checked for valid session number, learner ID, action code, policy code, difficulty level, task category, reward, accuracy, response time, time on task, and task completion.

Formula-derived variables, including gain score, psychological engagement total, self-regulated learning score, motivation score, and task accuracy percentage, were recalculated during quality control. Descriptive distributions were inspected for extreme values, but values were removed only if they exceeded possible ranges or contradicted the coding scheme.

### 2.9 Sample size and power considerations

A sensitivity power analysis was conducted for the final sample of 100 learners per group. With a two-sided alpha level of 0.05 and 80% power, the design could detect a moderate between-group effect size (*d* = 0.40) in student-level outcomes. Thus, the sample was adequate for detecting moderate primary outcome differences but less suitable for estimating complex high-dimensional RL policy effects or small mediation effects. For session-level analyses, 3,200 learner-session records were analyzed as nested observations, and mixed-effects models included learner-level random intercepts.

### 2.10 Statistical analysis

Descriptive statistics were calculated for baseline characteristics, English proficiency, RL variables, learning-log indicators, psychological outcomes, performance outcomes, and user-experience indicators. Continuous variables were summarized as means and standard deviations, and categorical variables as frequencies and percentages. Baseline comparability was assessed using independent-samples t tests, chi-square tests, and standardized mean differences.

The primary student-level analysis estimated the association between group assignment and post-intervention total psychological engagement. Because baseline psychological engagement was unavailable, this analysis was interpreted as an adjusted post-intervention group comparison rather than a within-person improvement analysis. The adjusted student-level model was specified in Equation 14:


$$Y_{i}=\beta_{0}+\beta_{1}G_{i}+\beta_{2}P_{i}+\beta_{3}E_{i}+\beta_{4}A_{i}+\beta_{5}X_{i}+\varepsilon_{i}$$ (14)

where $Y_{i}$ is the outcome for learner i , *Gᵢ* indicates assignment to the RL-personalized or control condition, $P_{i}$ is baseline English proficiency, $E_{i}$ is the prior English score, $A_{i}$ is learner age, $X_{i}$ represents categorical covariates including gender, academic level, and major, and $\varepsilon_{i}$ is the residual error term. Adjusted linear models were estimated for psychological engagement total, self-regulated learning score, motivation score, gain score, task accuracy percentage, final achievement, satisfaction, perceived personalization, and perceived feedback quality. Model outputs were reported numerically using unstandardized coefficients, standard errors, 95% confidence intervals, test statistics, *p*-values, and adjusted *p*-values where applicable. For interaction-level outcomes, repeated learner-session observations were analyzed using mixed-effects models with learner-level random intercepts. The main repeated-measures model was specified in Equation 15:


$$Y_{\mathrm{it}}=\beta_{0}+\beta_{1}G_{i}+\beta_{2}S_{t}+\beta_{3}(G_{i}\times S_{t})+u_{i}+\varepsilon_{\mathrm{it}}$$ (15)

where $Y_{\mathrm{it}}$ is the session-level outcome for learner i at session t , $G_{i}$ denotes study group, $S_{t}$ denotes session number, $G_{i}\times S_{t}$ estimates whether the trajectory differed between groups, $u_{i}$ is the learner-specific random intercept, and $\varepsilon_{\mathrm{it}}$ is the residual error. Session-level outcomes included reward value, task accuracy, response time, task completion, and time on task.

Momentary engagement was treated only as a process indicator and was not used to support claims about questionnaire-based psychological engagement. Model assumptions were assessed using residual histograms, Q-Q plots, Shapiro–Wilk tests, and residual-versus-fitted plots; robust standard errors or non-parametric sensitivity checks were considered when assumptions were materially violated. The primary outcome was tested at two-sided *α* = 0.05. For multiple secondary outcomes, Benjamini-Hochberg false-discovery-rate adjustment was applied by outcome family, with unadjusted *p*-values and adjusted *q*-values reported where applicable. Cohen’s *d* was used for continuous between-group differences, and chi-square statistics with Cramer’s *V* were used for categorical baseline variables. Effect sizes, particularly for self-report outcomes, were interpreted with caution given possible intervention, novelty, perceived personalization, demand characteristics, and unequal exposure effects. Pearson correlations among psychological, user-experience, and performance outcomes were calculated to assess construct overlap. Exploratory mechanism analyses examined whether adaptive process indicators, including difficulty exposure, reward, self-regulation prompts, scenario-based tasks, task completion, and time on task, partially explained group differences. Mediation results, where reported, were interpreted as exploratory and mechanism-consistent rather than definitive causal evidence. All tests were two-sided, with 95% confidence intervals. Table 8 summarizes the analysis plan.

**Table 8:** Statistical analysis and reporting plan.

Analytical objective	Data level	Model or statistic	Required reporting
Baseline comparability	Student-level	*t* test, Chi-square test, standardized mean difference	Group means/proportions, test statistic, *p*-value, standardized difference
Primary outcome		Adjusted linear model	*β*, SE, 95% CI, *t* value, *p*-value, effect size
Secondary psychological outcomes			
Performance outcomes			
User-experience outcomes			
Session-level trajectories	Interaction-level	Mixed-effects model with learner random intercept	Group effect, session effect, Group × Session interaction, SE, 95% CI, *p*-value
Residual assumptions	Model-level	Residual plots, Q-Q plots, Shapiro–Wilk test	Brief diagnostic summary
Multiple comparisons	Outcome-family level	Benjamini-Hochberg FDR	Unadjusted *p*-values and adjusted *q*-values
Construct overlap	Student-level	Pearson correlation matrix	Correlation coefficients among psychological, user-experience, and performance outcomes
Exploratory mechanisms	Student-level or interaction-level aggregates	Mediation or process models	Indirect estimates, bootstrap CI, cautious interpretation

### 2.11 Ethical considerations

The study involved human participants and was conducted in accordance with institutional procedures for research with anonymized educational data and the principles of the Declaration of Helsinki. Learners were informed about the purpose of the study, the voluntary nature of research participation, the use of anonymized learning and questionnaire data, and their right to decline research participation without academic penalty. Informed consent was obtained before inclusion of learner data in the research dataset. All records were anonymized before analysis using coded learner identifiers.

## 3 Results

### 3.1 Participant characteristics and baseline equivalence

The final analytic sample included 200 participants, with equal allocation to the RL-personalized group (*n* = 100) and the control group (*n* = 100). As shown in Table 9, the two groups were comparable in age, gender distribution, academic level, and major distribution, with no statistically significant baseline demographic differences. Baseline English proficiency was also balanced between groups. Prior English scores, overall pre-test English scores, and domain-specific pre-test scores for vocabulary, reading, writing, and speaking did not differ significantly between the RL-personalized and control groups. Baseline psychological engagement, self-regulated learning, and motivation were not measured; therefore, subsequent psychological comparisons were interpreted as adjusted post-intervention between-group differences rather than within-person change.

**Table 9:** Participant characteristics and baseline English proficiency equivalence between study groups.

Variable	Total (*n* = 200)	RL-personalized (*n* = 100)	Control (*n* = 100)	Mean difference	Test statistic	*p*-value
Sample size, *n* (%)	200 (100.0)	100 (50.0)	100 (50.0)	—	—	—
Age, years, mean ± SD	23.26 ± 2.92	23.48 ± 3.04	23.03 ± 2.79	0.45	*t* = 1.090	0.277
Gender, *n* (%)				—	*χ*^2^ = 0.516	0.773
Female	95 (47.5)	50 (50.0)	45 (45.0)	—	—	—
Male	99 (49.5)	47 (47.0)	52 (52.0)	—	—	—
Prefer not to say	6 (3.0)	3 (3.0)	3 (3.0)	—	—	—
Academic level, *n* (%)				—	*χ*^2^ = 4.398	0.111
Undergraduate	109 (54.5)	61 (61.0)	48 (48.0)	—	—	—
Master’s	70 (35.0)	28 (28.0)	42 (42.0)	—	—	—
Professional certificate	21 (10.5)	11 (11.0)	10 (10.0)	—	—	—
Major, *n* (%)				—	*χ*^2^ = 1.399	0.924
Business/Management	50 (25.0)	26 (26.0)	24 (24.0)	—	—	—
Engineering	42 (21.0)	20 (20.0)	22 (22.0)	—	—	—
Health sciences	21 (10.5)	11 (11.0)	10 (10.0)	—	—	—
Social sciences	32 (16.0)	18 (18.0)	14 (14.0)	—	—	—
Computer science	37 (18.5)	16 (16.0)	21 (21.0)	—	—	—
Other	18 (9.0)	9 (9.0)	9 (9.0)	—	—	—
Prior English score, mean ± SD	59.28 ± 10.30	59.49 ± 10.45	59.07 ± 10.19	0.42	*t* = 0.285	0.776
Pre-test English score, mean ± SD	58.35 ± 10.68	58.33 ± 10.71	58.37 ± 10.70	−0.04	*t* = −0.024	0.981
Vocabulary pre-test, mean ± SD	59.41 ± 11.10	59.26 ± 11.16	59.56 ± 11.09	−0.30	*t* = −0.190	0.849
Reading pre-test, mean ± SD	59.09 ± 11.13	59.32 ± 11.03	58.87 ± 11.28	0.45	*t* = 0.284	0.777
Writing pre-test, mean ± SD	56.87 ± 11.59	57.16 ± 11.47	56.58 ± 11.77	0.58	*t* = 0.354	0.724
Speaking pre-test, mean ± SD	58.04 ± 11.44	57.70 ± 11.88	58.38 ± 11.02	−0.68	*t* = −0.419	0.676

### 3.2 Reinforcement learning personalization and learning interaction patterns

**Figure 4:**
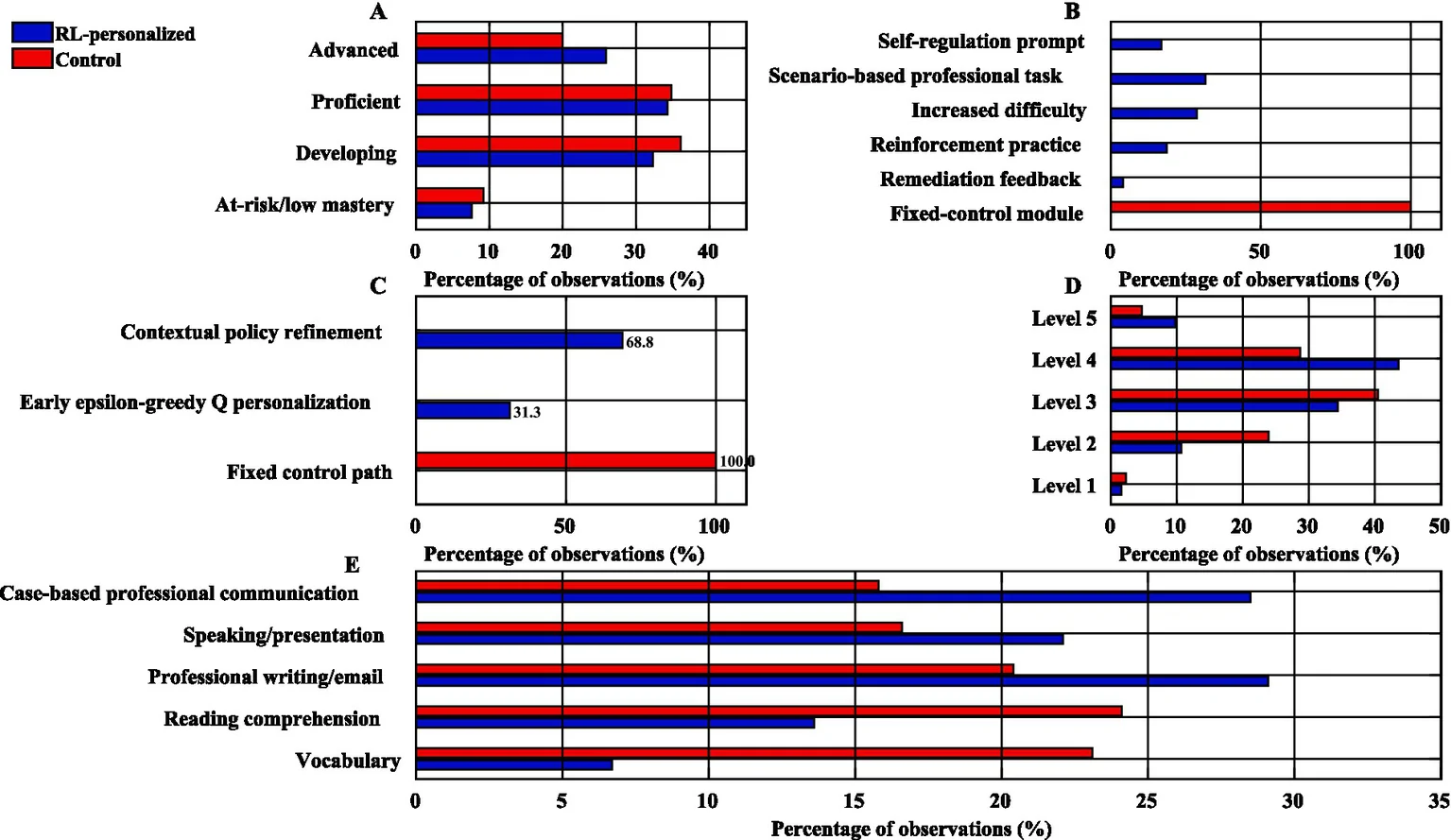
Distribution of learner states, instructional actions, policy types, task difficulty levels, and recommended task categories across session-level observations. **(A)** Learner states. **(B)** Instructional actions. **(C)** Policy types. **(D)** Task difficulty levels. **(E)** Recommended task categories.

As shown in Figure 4, the RL-personalized condition was characterized by a greater proportion of advanced learner states, higher use of increased-difficulty, scenario-based, and self-regulation-oriented instructional actions, and a stronger focus on contextual policy refinement. In contrast, the control condition remained fixed in instructional actions and policy structure. The RL-personalized group also showed a higher proportion of level 4 and level 5 tasks and a greater emphasis on professional writing/email, speaking/presentation, and case-based professional communication activities.

Student-level interaction indicators showed greater process-level learning activity in the RL-personalized group (Table 10). Compared with the control group, learners in the RL-personalized condition showed higher mean reward, higher mean difficulty exposure, greater login frequency, longer time on task, more completed tasks, more attempts, higher session task accuracy, and higher momentary engagement process scores, while showing shorter response times.

**Table 10:** Student-level learning-interaction process indicators by study group.

Indicator	RL-personalized (*n* = 100), mean ± SD	Control (*n* = 100), mean ± SD	Mean difference	*t* value	*p*-value
Mean reward	0.592 ± 0.172	0.334 ± 0.183	0.259	10.324	<0.001
Mean difficulty level	3.491 ± 0.562	3.097 ± 0.135	0.394	6.820	<0.001
Login frequency	20.100 ± 1.554	17.890 ± 1.317	2.210	10.849	<0.001
Time on task, min	589.188 ± 35.551	528.607 ± 38.741	60.581	11.522	<0.001
Completed tasks	159.440 ± 9.234	138.910 ± 10.121	20.530	14.986	<0.001
Attempts	196.330 ± 11.165	175.870 ± 11.841	20.460	12.571	<0.001
Average response time, sec	72.664 ± 2.989	76.612 ± 5.784	−3.948	−6.064	<0.001
Session task accuracy, %	82.790 ± 3.655	79.729 ± 5.244	3.061	4.789	<0.001
Momentary engagement process score	3.204 ± 0.454	2.886 ± 0.420	0.318	5.142	<0.001

**Figure 5:**
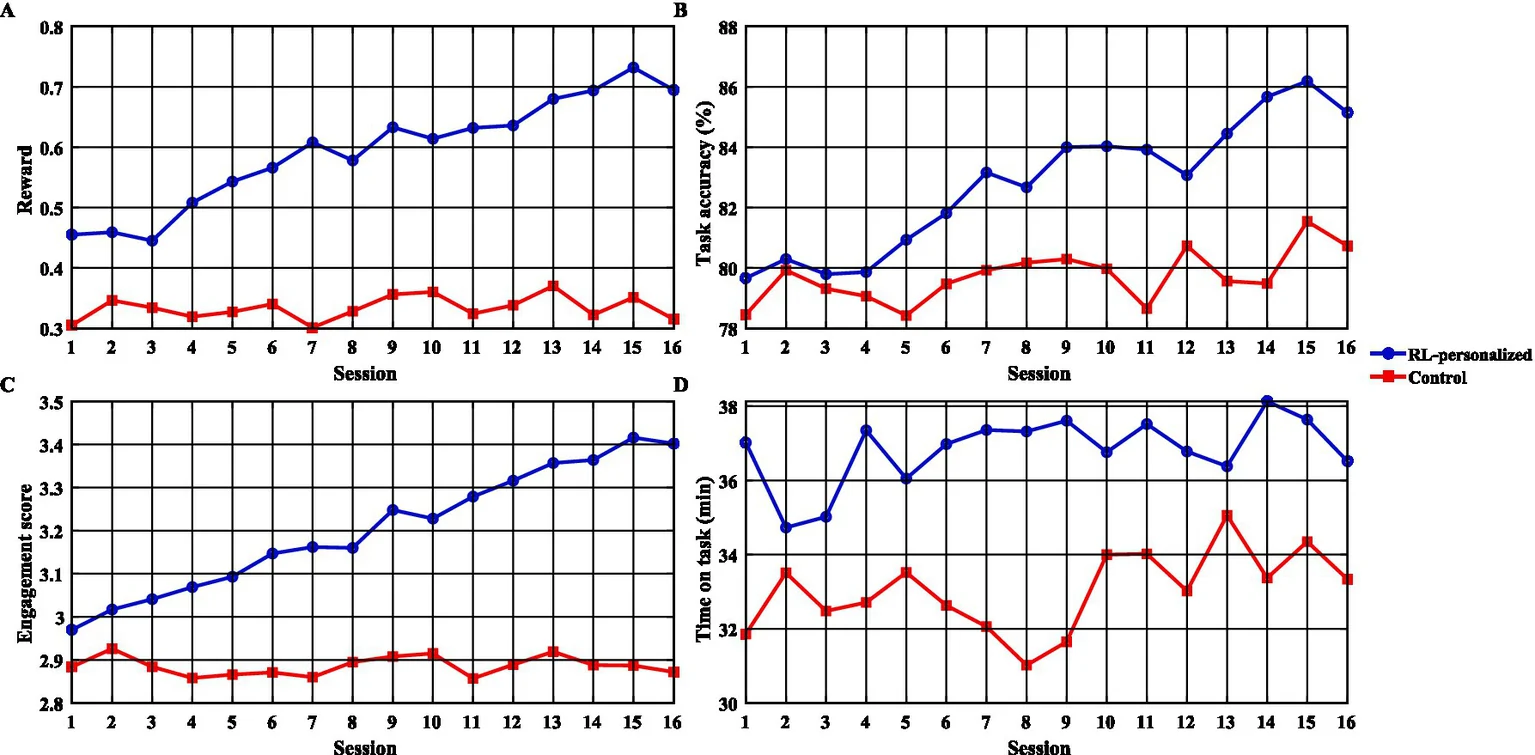
Session-level trajectories of reward, task accuracy, momentary engagement process score, and time on task across the 16 instructional sessions. **(A)** Reward values. **(B)** Task accuracy. **(C)** Momentary engagement process score. **(D)** Time on task.

Momentary engagement was treated as a session-level process indicator and was not used to substantiate claims about the primary questionnaire-based psychological engagement outcome. The session-level trajectories in Figure 5 showed a generally upward pattern for the RL-personalized group across reward, task accuracy, momentary engagement process score, and time on task over the 16 instructional sessions. In contrast, the control group displayed comparatively flatter trajectories in these process indicators.

### 3.3 Effects on psychological engagement

**Figure 6:**
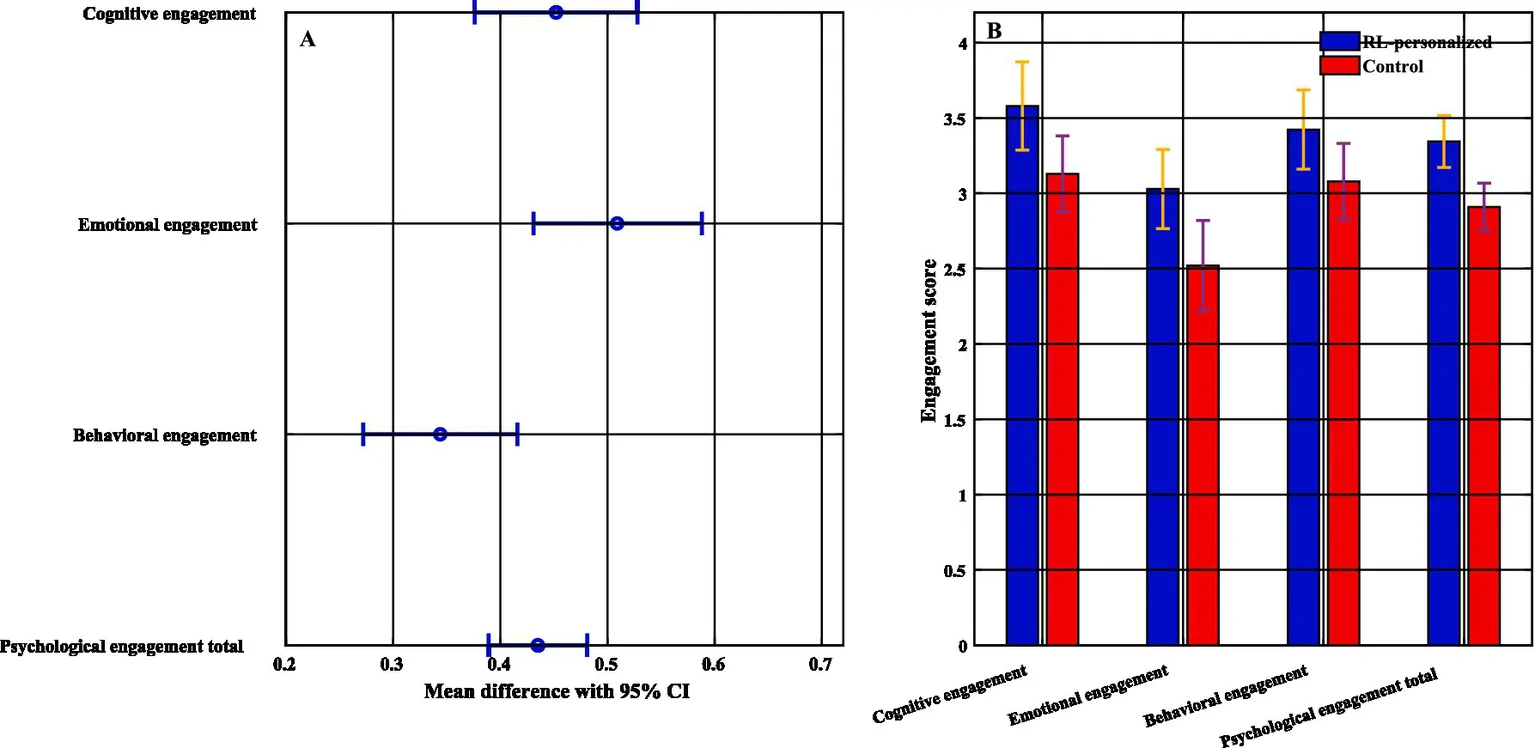
Psychological engagement outcomes by study group. **(A)** Mean differences with 95% confidence intervals. **(B)** Group means with standard deviations.

Figure 6 shows that the RL-personalized group showed higher post-intervention psychological engagement scores than the control group across all engagement dimensions. The largest between-group difference was observed for emotional engagement, followed by cognitive engagement, total psychological engagement, and behavioral engagement. Because baseline psychological engagement was not measured, these findings were interpreted as adjusted post-intervention between-group differences rather than within-person improvement.

As illustrated in Figure 6A, all mean differences were positive, and their 95% confidence intervals did not cross zero. Figure 6B shows higher mean scores for cognitive, emotional, behavioral, and total psychological engagement in the RL-personalized group, indicating post-intervention between-group differences favoring the RL-personalized condition (see Table 11).

**Table 11:** Post-intervention psychological engagement outcomes by study group.

Outcome	RL-personalized (*n* = 100), mean ± SD	Control (*n* = 100), mean ± SD	Mean difference	95% CI	*t* value	*p*-value	*q* value	Cohen’s *d*
Cognitive engagement	3.580 ± 0.293	3.129 ± 0.252	0.452	0.376 to 0.528	11.694	<0.001	<0.001	1.654
Emotional engagement	3.029 ± 0.263	2.520 ± 0.299	0.509	0.431 to 0.588	12.789	<0.001	<0.001	1.809
Behavioral engagement	3.423 ± 0.263	3.079 ± 0.252	0.344	0.272 to 0.416	9.442	<0.001	<0.001	1.335
Psychological engagement total	3.344 ± 0.173	2.909 ± 0.159	0.435	0.389 to 0.481	18.519	<0.001	<0.001	2.619

### 3.4 Effects on self-regulated learning and motivation

As shown in Table 12, the RL-personalized group showed higher post-intervention self-regulated learning and motivation outcomes than the control group. All self-regulation components, including goal setting, planning, monitoring, and revision behavior, favored the RL-personalized condition, with the overall self-regulated learning score showing a large between-group difference.

**Table 12:** Group differences in self-regulated learning and motivation outcomes.

Outcome	RL-personalized (*n* = 100), mean ± SD	Control (*n* = 100), mean ± SD	Mean difference	95% CI	*t* value	*p*-value	*q* value	Cohen’s *d*
Goal setting	3.428 ± 0.249	3.011 ± 0.263	0.417	0.346 to 0.488	11.530	<0.001	<0.001	1.631
Planning	3.271 ± 0.278	2.923 ± 0.293	0.349	0.269 to 0.428	8.631	<0.001	<0.001	1.221
Monitoring	3.446 ± 0.279	3.029 ± 0.281	0.418	0.340 to 0.496	10.554	<0.001	<0.001	1.493
Revision behavior	3.362 ± 0.275	3.050 ± 0.276	0.312	0.235 to 0.389	8.004	<0.001	<0.001	1.132
Self-regulated learning score	3.377 ± 0.160	3.003 ± 0.159	0.374	0.329 to 0.418	16.580	<0.001	<0.001	2.345
Intrinsic motivation	3.623 ± 0.231	3.188 ± 0.270	0.435	0.365 to 0.505	12.238	<0.001	<0.001	1.731
Learning interest	3.568 ± 0.239	3.062 ± 0.229	0.507	0.441 to 0.572	15.307	<0.001	<0.001	2.165
Perceived usefulness	3.209 ± 0.271	2.817 ± 0.227	0.393	0.323 to 0.462	11.104	<0.001	<0.001	1.570
Motivation score	3.467 ± 0.153	3.022 ± 0.149	0.445	0.403 to 0.487	20.853	<0.001	<0.001	2.949

Motivational outcomes showed a similar post-intervention pattern. Intrinsic motivation, learning interest, perceived usefulness, and total motivation score were higher in the RL-personalized group. The largest effect was observed for the total motivation score, followed by the self-regulated learning score and learning interest.

### 3.5 Effects on English learning performance

**Figure 7:**
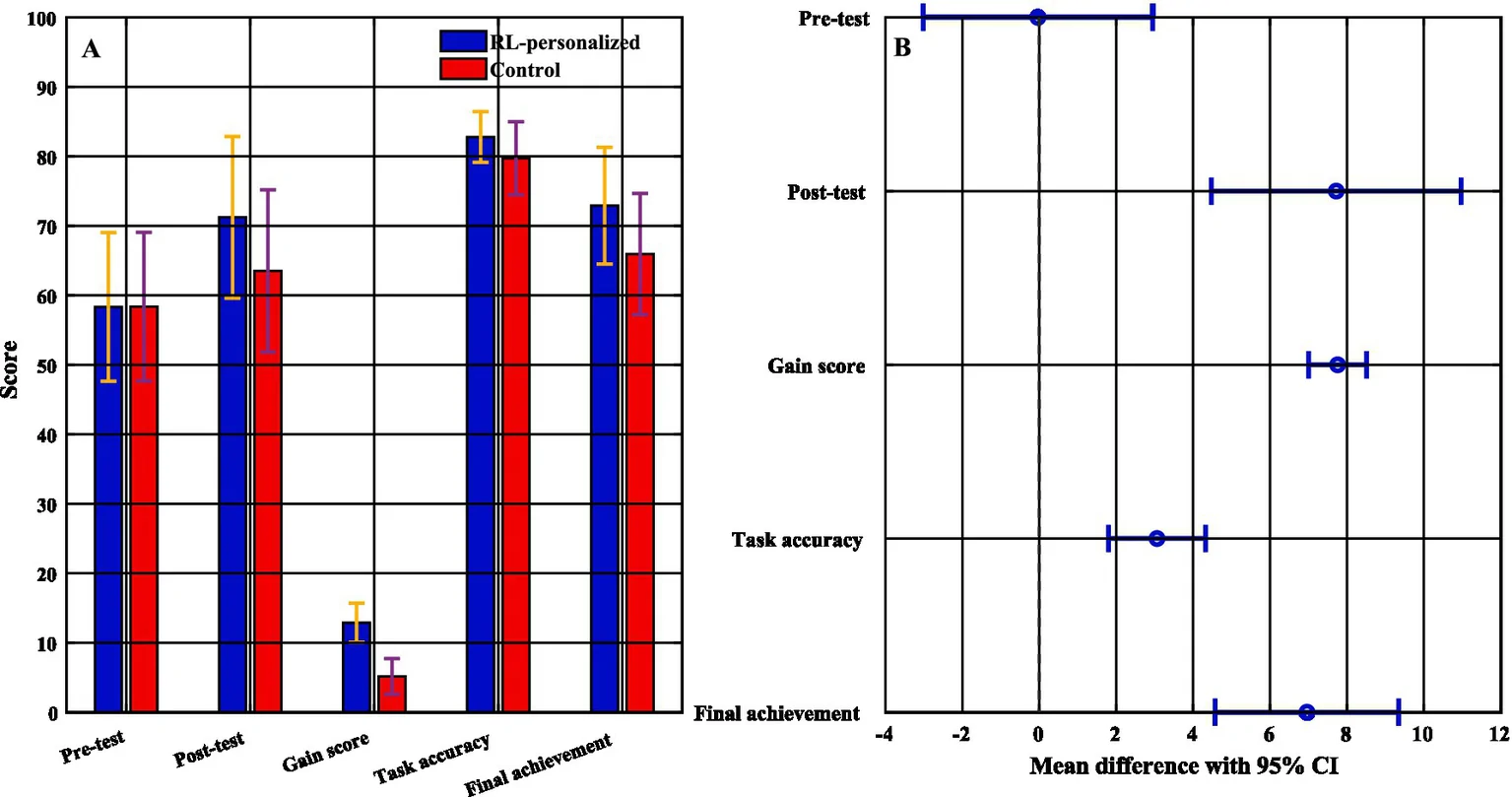
English learning performance outcomes by study group. **(A)** Group means with standard deviations. **(B)** Mean differences with 95% confidence intervals.

Figure 7 shows that baseline English performance was comparable between the RL-personalized and control groups, with no meaningful difference in pre-test scores. Post-intervention outcomes favored the RL-personalized group for post-test score, gain score, task accuracy, and final achievement.

The mean-difference estimates in Figure 7B show that the largest between-group difference was observed for gain score, followed by post-test score and final achievement. Task accuracy also favored the RL-personalized group, whereas the pre-test confidence interval crossed zero, confirming baseline equivalence before the intervention. Overall, the findings indicate higher post-intervention English learning performance in the RL-personalized condition across achievement and task-based indicators.

### 3.6 User experience and perceived personalization

As shown in Table 13, the RL-personalized group reported higher post-intervention user-experience outcomes than the control group. Satisfaction, perceived personalization, and perceived feedback quality all favored the RL-personalized condition.

**Table 13:** Group differences in user experience and perceived personalization outcomes.

User-experience outcome	RL-personalized (*n* = 100), mean ± SD	Control (*n* = 100), mean ± SD	Mean difference	95% CI	*t* value	*p*-value	*q* value	Cohen’s *d*
Satisfaction	3.977 ± 0.246	3.375 ± 0.251	0.602	0.532 to 0.671	17.116	<0.001	<0.001	2.421
Perceived personalization	3.676 ± 0.338	2.720 ± 0.341	0.956	0.861 to 1.050	19.906	<0.001	<0.001	2.815
Perceived feedback quality	3.674 ± 0.283	3.001 ± 0.288	0.673	0.594 to 0.753	16.690	<0.001	<0.001	2.360

Perceived personalization showed the largest between-group difference, followed by perceived feedback quality and satisfaction. Because these were self-report outcomes and several effect sizes were large, the estimates were interpreted as post-intervention between-group differences that may reflect adaptive instruction, novelty, perceived personalization, demand characteristics, and differential instructional exposure.

### 3.7 Correlations among psychological, user-experience, and performance outcomes

Pearson correlations among psychological, user-experience, and performance outcomes are shown in Table 14. Psychological engagement, self-regulated learning, motivation, and user-experience indicators were moderately to strongly correlated, while their correlations with performance outcomes were generally smaller except for gain score. Post-test score and final achievement were almost perfectly correlated, indicating substantial overlap between these two performance indicators.

**Table 14:** Correlation matrix among psychological, user-experience, and performance outcomes.

Variable	1	2	3	4	5	6	7	8	9
1. Psychological engagement total	1.000	0	0	0	0	0	0	0	0
2. Self-regulated learning score	0.700	1.000	0	0	0	0	0	0	0
3. Motivation score	0.722	0.642	1.000	0	0	0	0	0	0
4. Satisfaction	0.705	0.588	0.666	1.000	0	0	0	0	0
5. Perceived personalization	0.705	0.692	0.675	0.648	1.000	0	0	0	0
6. Perceived feedback quality	0.582	0.633	0.646	0.600	0.647	1.000	0	0	0
7. Post-test score	0.442	0.218	0.323	0.298	0.262	0.226	1.000	0	0
8. Gain score	0.688	0.602	0.684	0.624	0.673	0.608	0.504	1.000	0
9. Final achievement	0.517	0.274	0.380	0.352	0.318	0.274	0.995	0.538	1.000

Values are Pearson correlation coefficients; *n* = 200. All reported correlations were statistically significant at *p* < 0.01.

### 3.8 Adjusted and repeated-measures model results

**Figure 8:**
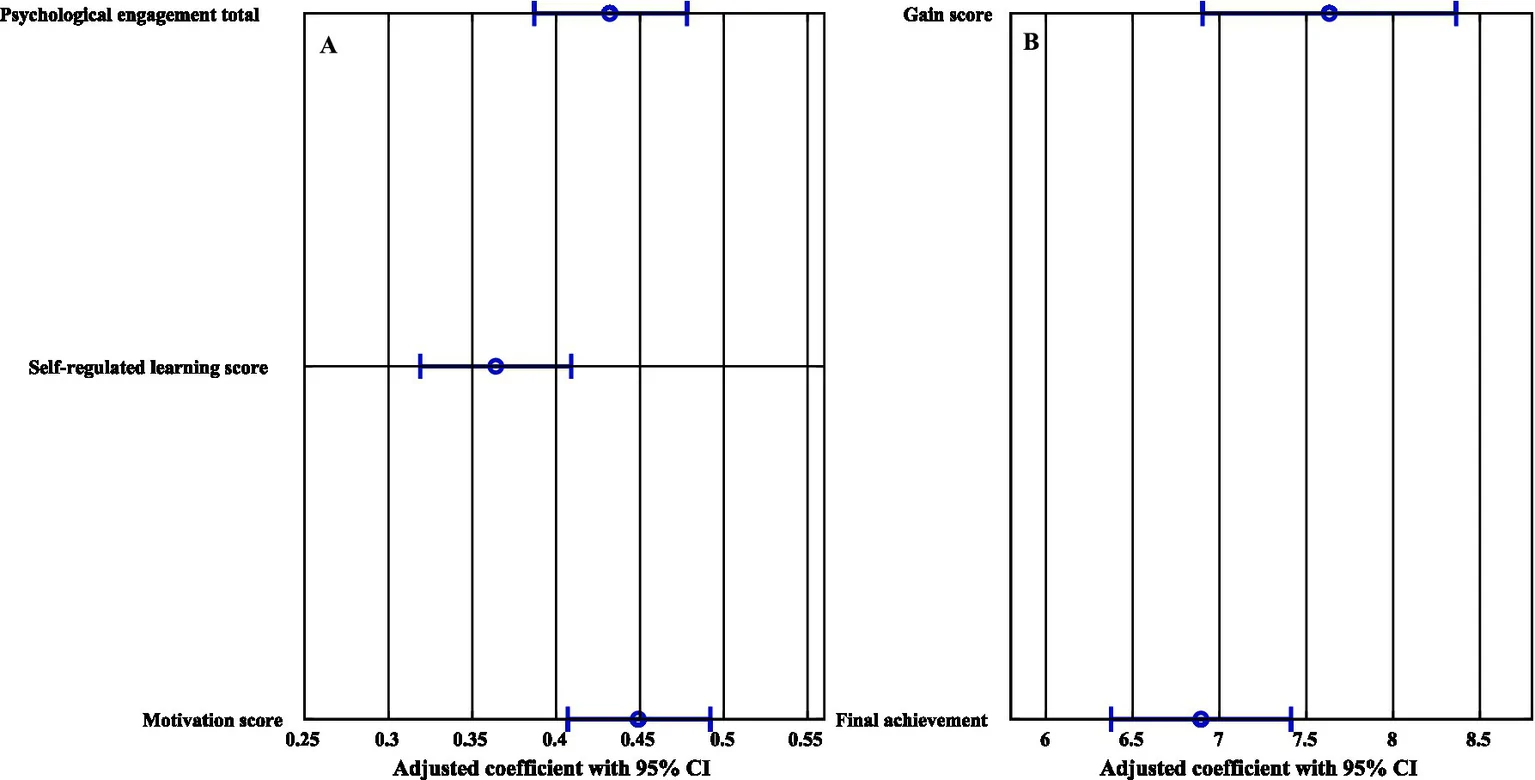
Adjusted group coefficients for psychological and learning-performance outcomes. **(A)** Psychological outcomes. **(B)** Learning-performance outcomes.

Figure 8 presents adjusted group coefficients for psychological and learning-performance outcomes. After adjustment for baseline English proficiency, prior English score, age, gender, academic level, and major, the RL-personalized condition remained positively associated with psychological engagement total, self-regulated learning score, motivation score, gain score, and final achievement (Table 15).

**Table 15:** Adjusted student-level model results.

Outcome	*β*	SE	95% CI	*t* value	*p*-value	*q* value
Psychological engagement total	0.432	0.023	0.387 to 0.478	18.839	<0.001	<0.001
Self-regulated learning score	0.364	0.023	0.319 to 0.409	15.934	<0.001	<0.001
Motivation score	0.449	0.022	0.407 to 0.492	20.781	<0.001	<0.001
Gain score	7.633	0.370	6.903 to 8.363	20.630	<0.001	<0.001
Final achievement	6.893	0.263	6.375 to 7.412	26.225	<0.001	<0.001

Mixed-effects models for session-level process indicators showed Group × Session associations for reward value, task accuracy, and momentary engagement process score, while the Group × Session term for time on task was not statistically significant (Table 16). Momentary engagement remained interpreted only as a process indicator, not as evidence for the primary questionnaire-based psychological engagement outcome.

**Table 16:** Mixed-effects model results for session-level process indicators.

Outcome	Group *β*	Session *β*	Group × session *β*	SE	95% CI for group × session	*z* value	*p*-value	*q* value
Reward value	0.129	0.001	0.017	0.001	0.014 to 0.020	12.175	<0.001	<0.001
Task accuracy, %	0.629	0.109	0.324	0.047	0.232 to 0.417	6.892	<0.001	<0.001
Time on task, min	3.916	0.109	−0.017	0.057	−0.129 to 0.094	−0.304	0.761	0.761
Momentary engagement process score	0.096	0.000	0.030	0.002	0.026 to 0.033	15.861	<0.001	<0.001

Residual diagnostics showed no material violations requiring changes to the primary model specification. A minor residual-normality deviation in one adjusted model was checked using robust standard errors, and the substantive conclusions were unchanged.

### 3.9 Exploratory process analysis

Exploratory process models examined whether adaptive learning indicators were consistent with possible pathways linking group assignment to psychological engagement and learning gain. Models were adjusted for baseline English proficiency, prior English score, age, gender, academic level, and major. Mean reward value and task completion showed positive indirect/process estimates for both psychological engagement total and gain score, with bootstrap confidence intervals not crossing zero (Table 17). Time on task showed positive but weaker process estimates, whereas mean difficulty exposure, self-regulation-prompt exposure, and scenario-based-task exposure were not supported as standalone indirect pathways after adjustment.

**Table 17:** Exploratory process-analysis summary.

Process indicator	Outcome	Indirect/Process estimate	Bootstrap 95% CI	*p*-value	*q* value
Mean difficulty exposure	Psychological engagement total	−0.003	−0.037 to 0.029	0.804	0.804
Mean difficulty exposure	Gain score	0.071	−0.372 to 0.526	0.782	0.804
Mean reward value	Psychological engagement total	0.085	0.041 to 0.132	<0.001	<0.001
Mean reward value	Gain score	0.894	0.230 to 1.574	0.006	0.026
Self-regulation-prompt exposure	Psychological engagement total	0.027	−0.038 to 0.094	0.454	0.739
Self-regulation-prompt exposure	Gain score	0.145	−0.727 to 1.090	0.748	0.804
Scenario-based-task exposure	Psychological engagement total	0.018	−0.058 to 0.093	0.658	0.804
Scenario-based-task exposure	Gain score	0.369	−0.712 to 1.515	0.492	0.739
Task completion	Psychological engagement total	0.075	0.024 to 0.124	0.003	0.017
Task completion	Gain score	0.935	0.202 to 1.640	0.014	0.043
Time on task	Psychological engagement total	0.034	−0.005 to 0.073	0.085	0.170
Time on task	Gain score	0.516	−0.032 to 1.033	0.066	0.159

These analyses were interpreted as exploratory and mechanism-consistent rather than as definitive causal mediation evidence, because process indicators were not independently randomized and may reflect multiple features of the RL-personalized pathway.

## 4 Discussion

This study examined an RL-personalized Professional English instructional pathway in relation to psychological engagement, self-regulated learning, motivation, user experience, learning-interaction patterns, and English performance. The revised findings indicate that the RL-personalized condition was associated with higher post-intervention psychological, self-regulatory, motivational, user-experience, and performance outcomes than the fixed-path control condition. Baseline demographic characteristics and English proficiency were comparable between groups, supporting interpretation of the findings as adjusted post-intervention group differences. However, because baseline levels of psychological engagement, self-regulated learning, and motivation were not measured, the psychological findings should not be interpreted as within-person improvements in these constructs.

A central finding was that the RL-personalized condition showed different learner–system interaction patterns from the fixed-path control condition. Learners in the RL-personalized group were more frequently exposed to higher difficulty levels, scenario-based professional tasks, self-regulation prompts, and contextual policy refinement. This pattern is consistent with the theoretical function of reinforcement learning in education: adaptive systems can update instructional decisions in response to learner states, prior actions, and reward signals rather than delivering a fixed instructional sequence (Maghsudi et al., 2021; Den Hengst et al., 2020; Memarian and Doleck, 2024). In the present study, this adaptive logic appeared in the distribution of policy types and instructional actions, as well as in session-level trajectories of reward, task accuracy, momentary engagement process score, and time on task. These process findings are consistent with progressive adaptation over the instructional period. However, they do not isolate the Q-learning algorithm’s independent causal effect from the broader adaptive instructional pathway.

The findings on psychological engagement should be interpreted in this context. Learners in the RL-personalized group reported higher post-intervention cognitive, emotional, behavioral, and total psychological engagement than those in the control group. Engagement in technology-enhanced learning is multidimensional and includes cognitive investment, emotional involvement, and behavioral participation. Recent learning analytics research has emphasized that many digital learning studies rely heavily on behavioral traces such as clicks, time on task, or task completion. In contrast, fewer studies integrate psychological dimensions of engagement (Bergdahl et al., 2024). The present study addresses this limitation by combining learning-log indicators with questionnaire-based multidimensional engagement outcomes. Because the reward function did not include the post-intervention psychological engagement questionnaire, the primary engagement outcome remained analytically distinct from the algorithmic reward signal.

The results also showed higher post-intervention self-regulated learning in the RL-personalized group, including goal setting, planning, monitoring, and revision behavior. This pattern is consistent with evidence that learning analytics and adaptive feedback systems can support learners’ regulatory processes by making learning progress visible and by guiding strategic adjustment (Heikkinen et al., 2023). Within the present pathway, self-regulation prompts may have helped learners monitor their performance, adjust their effort, and revise their learning strategies. This interpretation aligns with meta-analytic evidence showing that self-regulated learning interventions are associated with better learning strategies and academic performance among university students (Theobald, 2021). Therefore, the RL-personalized pathway may have functioned not only as a task-sequencing system but also as a regulatory scaffold.

Motivation outcomes followed a similar post-intervention pattern. Intrinsic motivation, learning interest, perceived usefulness, and total motivation scores were higher in the RL-personalized group. This finding is consistent with AI-supported personalized education, in which adaptive pathways may increase perceived relevance by matching learning tasks to learners’ needs, weaknesses, and progression states (Maghsudi et al., 2021). In Professional English education, perceived relevance is especially important because learners often evaluate tasks according to their applicability to workplace, academic, or professional communication demands. The greater exposure to professional writing/email, speaking/presentation, and case-based communication tasks in the RL-personalized condition may therefore have supported perceived usefulness and learning interest. At the same time, this interpretation should remain cautious. Although RL uses reward signals, these signals were internal to the instructional algorithm and were not presented to learners as external controlling incentives. Learners experienced the system through adaptive difficulty, task relevance, and feedback, which may explain why intrinsic motivation was higher rather than lower.

The performance findings were consistent with the psychological and process-level results. Although pre-test English performance was comparable between groups, post-intervention outcomes favored the RL-personalized group for post-test score, gain score, task accuracy, and final achievement. These findings are consistent with recent evidence from blended EFL learning in which analytics-based feedback was associated with self-regulated learning and academic achievement (Chen, 2023). In the present study, the largest performance difference was observed for the gain score, suggesting that the RL-personalized condition was associated with greater change in English proficiency from baseline. However, this should be understood as a group-associated learning-performance difference within the implemented pathway, not as proof that Q-learning alone produced the observed gain.

The user-experience findings provide additional evidence regarding acceptability and perceived instructional quality. Learners in the RL-personalized group reported higher satisfaction, perceived personalization, and perceived feedback quality. The largest effect was observed for perceived personalization, suggesting that learners recognized the adaptive nature of the instructional pathway. Prior learning analytics research has shown that personalized feedback is more likely to support engagement when learners receive actionable, individualized guidance rather than generic information (Karaoglan Yilmaz and Yilmaz, 2022). The present findings extend this idea to a Professional English context by showing that learner-state-based adaptation and feedback were associated with both process-level differences and higher perceived instructional responsiveness.

The adjusted and exploratory analyses further clarify the interpretation of the findings. Adjusted student-level models showed that the RL-personalized condition remained positively associated with psychological engagement, self-regulated learning, motivation, gain score, and final achievement after controlling for baseline English proficiency and participant-level covariates. Mixed-effects models also indicated Group × Session associations for several session-level process indicators. In addition, the exploratory process analysis suggested that the mean reward value and task completion were mechanism-consistent indicators of psychological engagement and the gain score. These findings support a plausible pathway in which adaptive task selection, interaction quality, and sustained task completion are linked with psychological and performance outcomes. However, these analyses were exploratory and should not be interpreted as definitive evidence of causal mediation because the process indicators were not independently randomized.

The correlation matrix also informs the interpretation of psychological and user-experience outcomes. Psychological engagement, self-regulated learning, motivation, satisfaction, perceived personalization, and perceived feedback quality were moderately to strongly correlated, indicating that these constructs were related but not fully redundant. Their correlations with performance outcomes were generally smaller, except for gain score, suggesting that psychological and user-experience outcomes captured partially distinct dimensions of the learning process. The very high correlation between post-test score and final achievement indicates substantial overlap between these two performance indicators and should be considered when interpreting the performance domain.

The theoretical contribution of the study is therefore best framed as an applied, mechanism-informed contribution rather than as definitive evidence for a new causal theory of RL-personalized learning. The existing RL-in-education literature has emphasized RL’s potential to sequence content, select interventions, and optimize learning pathways (Den Hengst et al., 2020; Memarian and Doleck, 2024). The present study extends this work by linking a constrained RL-personalized pathway with psychological engagement, self-regulated learning, motivation, user experience, and performance indicators in Professional English education. A coherent interpretation is that adaptive difficulty may have supported challenge-skill alignment; self-regulation prompts may have supported planning and monitoring; professionally relevant tasks may have supported perceived usefulness and interest; and feedback may have supported perceived competence and continued engagement. These mechanisms remain plausible interpretations rather than established causal pathways.

The findings have practical implications for Professional English programs. First, adaptive task sequencing may be useful in courses where learners differ in prior proficiency, professional goals, and communication needs. Second, adaptive systems should incorporate psychological and self-regulatory indicators alongside performance metrics, because optimizing only for short-term accuracy may neglect persistence, effort regulation, engagement, or perceived relevance. Third, adaptive feedback should remain interpretable and pedagogically actionable. The higher perceived personalization and feedback-quality scores suggest that learners may respond more favorably when recommendations are experienced as relevant, supportive, and aligned with professional communication needs.

Several limitations should be considered. First, although baseline equivalence was established for demographic characteristics and English proficiency, baseline psychological engagement, self-regulated learning, and motivation were not measured. Therefore, the psychological findings represent adjusted post-intervention group differences rather than within-person changes. Second, the RL-personalized condition differed from the control condition not only in the decision algorithm but also in difficulty exposure, task sequencing, scenario-based tasks, self-regulation prompts, feedback, time on task, and task completion. Consequently, the study cannot determine whether the observed differences were attributable specifically to Q-learning, to personalization more broadly, or to differential instructional exposure. Third, several outcomes were self-reported, and some effect sizes were large; these estimates may partly reflect novelty effects, demand characteristics, perceived personalization, or response tendencies in addition to instructional differences. Fourth, the intervention lasted 8 weeks and did not include delayed follow-up, so the durability of psychological and performance differences remains uncertain. Fifth, the process analysis was exploratory and does not establish definitive causal mediation. Finally, RL-personalized systems require careful attention to transparency, algorithmic bias, data privacy, fairness, and learner autonomy, particularly when adaptive recommendations influence instructional pathways (Maghsudi et al., 2021; Memarian and Doleck, 2024).

This study suggests that an RL-personalized Professional English pathway was associated with higher post-intervention psychological engagement, self-regulated learning, motivation, user experience, and English performance than a fixed-path control pathway. The findings support the value of adaptive language-learning systems that combine learner-state estimation, difficulty calibration, professional task relevance, feedback, and self-regulation support within a single instructional pathway. However, the results should be interpreted as evidence for the implemented RL-personalized pathway rather than as isolated causal evidence for the Q-learning algorithm alone.

Several qualifications are necessary. Baseline psychological engagement, self-regulated learning, and motivation were not measured; therefore, the psychological findings represent adjusted post-intervention between-group differences rather than within-person improvement. In addition, the personalized condition differed from the control condition in multiple instructional features, including difficulty exposure, task sequencing, feedback, self-regulation prompts, time on task, and task completion. Self-report outcomes and large effect sizes should also be interpreted with caution, as novelty, perceived personalization, demand characteristics, and differential exposure may have contributed to the observed differences.

Future research should replicate these findings across broader Professional English contexts, include baseline psychological measures and delayed follow-up assessments, and use designs that can separate the contribution of the RL decision policy from related adaptive instructional features. Further work should also compare alternative reward functions, policy structures, and levels of instructor oversight, while examining how learners interpret and respond to adaptive recommendations.

## Data Availability

The original contributions presented in the study are included in the article/supplementary material, further inquiries can be directed to the corresponding author.

## References

[ref1] Bedenlier S. Bond M. Buntins K. Zawacki-Richter O. Kerres M. (2020). Facilitating student engagement through educational technology in higher education: a systematic review in the field of arts and humanities. Australas. J. Educ. Technol. 36, 126–150. doi: 10.14742/ajet.5477

[ref2] Bergdahl N. Bond M. Sjöberg J. Dougherty M. Oxley E. (2024). Unpacking student engagement in higher education learning analytics: a systematic review. Int. J. Educ. Technol. High. Educ. 21:63. doi: 10.1186/s41239-024-00493-y

[ref3] Bond M. Buntins K. Bedenlier S. Zawacki-Richter O. Kerres M. (2020). Mapping research in student engagement and educational technology in higher education: a systematic evidence map. Int. J. Educ. Technol. High. Educ. 17:2. doi: 10.1186/s41239-019-0176-8

[ref4] Chen J. (2023). Effects of learning analytics-based feedback on students’ self-regulated learning and academic achievement in an undergraduate EFL course. SSRN J. doi: 10.2139/ssrn.4589867

[ref5] Chen L. Chen P. Lin Z. (2020). Artificial intelligence in education: a review. IEEE Acc. 8, 75264–75278. doi: 10.1109/ACCESS.2020.2988510

[ref6] Cheng Z. Zhang Z. Xu Q. Maeda Y. Gu P. (2025). A meta-analysis addressing the relationship between self-regulated learning strategies and academic performance in online higher education. J. Comput. High. Educ. 37, 195–224. doi: 10.1007/s12528-023-09390-1

[ref7] Den Hengst F. Grua E. M. el Hassouni A. Hoogendoorn M. (2020). Reinforcement learning for personalization: a systematic literature review. Data Sci. 3, 107–147. doi: 10.3233/DS-200028

[ref8] Hasumi T. Chiu M.-S. (2024). Technology-enhanced language learning in English language education: performance analysis, core publications, and emerging trends. Cogent Educ. 11:2346044. doi: 10.1080/2331186X.2024.2346044

[ref9] Heikkinen S. Saqr M. Malmberg J. Tedre M. (2023). Supporting self-regulated learning with learning analytics interventions–a systematic literature review. Educ. Inf. Technol. 28, 3059–3088. doi: 10.1007/s10639-022-11281-4

[ref10] Karaoglan Yilmaz F. G. Yilmaz R. (2022). Learning analytics intervention improves students’ engagement in online learning. Technol. Knowl. Learn. 27, 449–460. doi: 10.1007/s10758-021-09547-w

[ref11] Maghsudi S. Lan A. Xu J. van der Schaar M. (2021). Personalized education in the artificial intelligence era: what to expect next. IEEE Signal Process. Mag. 38, 37–50. doi: 10.1109/MSP.2021.3055032

[ref12] Major L. Francis G. A. Tsapali M. (2021). The effectiveness of technology-supported personalised learning in low-and middle-income countries: a meta-analysis. Br. J. Educ. Technol. 52, 1935–1964. doi: 10.1111/bjet.13116

[ref13] Martin F. Chen Y. Moore R. L. Westine C. D. (2020). Systematic review of adaptive learning research designs, context, strategies, and technologies from 2009 to 2018. Educ. Technol. Res. Dev. 68, 1903–1929. doi: 10.1007/s11423-020-09793-2

[ref14] Memarian B. Doleck T. (2024). A scoping review of reinforcement learning in education. Comput. Educ. Open 6:100175. doi: 10.1016/j.caeo.2024.100175

[ref15] Qiao H. Zhao A. (2023). Artificial intelligence-based language learning: illuminating the impact on speaking skills and self-regulation in Chinese EFL context. Front. Psychol. 14:1255594. doi: 10.3389/fpsyg.2023.1255594, 38022973 PMC10652775

[ref16] Rahmati J. Izadpanah S. Shahnavaz A. (2021). A meta-analysis on educational technology in English language teaching. Lang. Test. Asia 11:7. doi: 10.1186/s40468-021-00121-w

[ref17] Seyyedrezaei M. S. Amiryousefi M. Gimeno-Sanz A. Tavakoli M. (2024). A meta-analysis of the relative effectiveness of technology-enhanced language learning on ESL/EFL writing performance: retrospect and prospect. Comput. Assist. Lang. Learn. 37, 1771–1805. doi: 10.1080/09588221.2022.2118782

[ref18] Shemshack A. Spector J. M. (2020). A systematic literature review of personalized learning terms. Smart Learn. Environ. 7:33. doi: 10.1186/s40561-020-00140-9

[ref19] Theobald M. (2021). Self-regulated learning training programs enhance university students’ academic performance, self-regulated learning strategies, and motivation: a meta-analysis. Contemp. Educ. Psychol. 66:101976. doi: 10.1016/j.cedpsych.2021.101976

[ref20] Xie H. Chu H.-C. Hwang G.-J. Wang C.-C. (2019). Trends and development in technology-enhanced adaptive/personalized learning: a systematic review of journal publications from 2007 to 2017. Comput. Educ. 140:103599. doi: 10.1016/j.compedu.2019.103599

[ref21] Zafari M. Bazargani J. S. Sadeghi-Niaraki A. Choi S.-M. (2022). Artificial intelligence applications in K−12 education: a systematic literature review. IEEE Acc. 10, 61905–61921. doi: 10.1109/ACCESS.2022.3179356

[ref22] Zawacki-Richter O. Marín V. I. Bond M. Gouverneur F. (2019). Systematic review of research on artificial intelligence applications in higher education–where are the educators? Int. J. Educ. Technol. High. Educ. 16:39. doi: 10.1186/s41239-019-0171-0

[ref23] Zhai X. Chu X. Chai C. S. Jong M. S. Y. Istenic A. Spector M. . (2021). A review of artificial intelligence (AI) in education from 2010 to 2020. Complexity 2021:8812542. doi: 10.1155/2021/8812542

